# Illegal hunting cases detected with molecular forensics in Brazil

**DOI:** 10.1186/2041-2223-3-17

**Published:** 2012-08-03

**Authors:** Alexandra Sanches, Paola M Tokumoto, Wellington AM Peres, Fernando L Nunes, Mariana ST Gotardi, Carolina S Carvalho, Cristiane Pelizzon, Tamissa G Godoi, Mauro Galetti

**Affiliations:** 1 Departamento de Ecologia, UNESP, CP 199, CEP 13506–900, Rio Claro, SP, Brazil; 2 Instituto Brasileiro do Meio Ambiente e dos Recursos Naturais Renováveis (IBAMA), CEP 78640–000, Canarana, MT, Brazil; 3 Instituto Brasileiro do Meio Ambiente e dos Recursos Naturais Renováveis (IBAMA), CEP 79002–380, Campo Grande, MS, Brazil

**Keywords:** Species identification, mtDNA, Wildlife forensics, Bush meat, Poaching, Neotropical region

## Abstract

**Background:**

Illegal hunting is one of the major threats to vertebrate populations in tropical regions. This unsustainable practice has serious consequences not only for the target populations, but also for the dynamics and structure of tropical ecosystems. Generally, in cases of suspected illegal hunting, the only evidence available is pieces of meat, skin or bone. In these cases, species identification can only be reliably determined using molecular technologies. Here, we reported an investigative study of three cases of suspected wildlife poaching in which molecular biology techniques were employed to identify the hunted species from remains of meat.

**Findings:**

By applying cytochrome *b* (cyt-*b*) and cytochrome oxidase subunit I (COI) molecular markers, the suspected illegal poaching was confirmed by the identification of three wild species, capybara (*Hydrochoerus hydrochaeris*), Chaco Chachalaca (*Ortalis canicollis*) and Pampas deer (*Ozotoceros bezoarticus*). In Brazil, hunting is a criminal offense, and based on this evidence, the defendants were found guilty and punished with fines; they may still be sentenced to prison for a period of 6 to 12 months.

**Conclusions:**

The genetic analysis used in this investigative study was suitable to diagnose the species killed and solve these criminal investigations. Molecular forensic techniques can therefore provide an important tool that enables local law enforcement agencies to apprehend illegal poachers.

## Findings

It is estimated that illegal hunting kills millions of vertebrates per year in tropical rainforests [1]. A number of reports have shown that the volume of wild game harvested is unsustainable and has led to the local extinction of several populations [1,2]. The use of wild foods or the bush meat crisis is one of the major challenges for the conservation of large-bodied vertebrates. Poor local law enforcement and corruption allow the hunting of large vertebrates to continue to be widespread even in protected areas [2].

Even when hunters are captured, the precise identification of bush meat is often questionable. Moreover, the remains of meat, fur, skin and bone are the only evidence recovered from the crime scene. In such cases, species identification can only be resolved with molecular tools [3-5].

**Figure 1:**
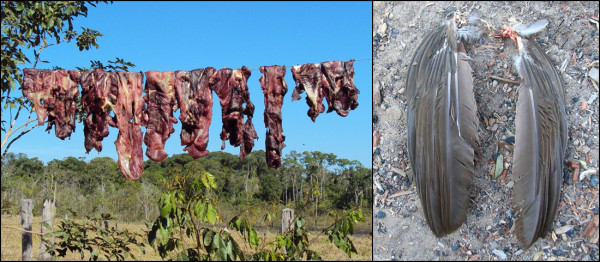
**Evidence of hunting.** Biltong of a mammal species (MAM1) (left) and wings remains (BIRD) of a bird species (right), both seized in the central-western Brazil region (cases 1 and 2).

Here, we reported an investigative study of three suspected offenses of wildlife poaching. In July 2010, based on suspected illegal hunting, a wildlife inspector of the Brazilian Environmental Agency (IBAMA) seized and sent us biltong samples of a mammal species (MAM1) (Figure 1). According to the inspector, the suspect claimed that the meat was pork, but there was no evidence to confirm this assertion (case 1). Along with MAM1, we received a second meat sample, which was removed from the wings of an unidentified bird species (BIRD; case 2) (Figure 1). In February 2011, we received a mammal meat sample (MAM2) taken by another wildlife inspector of IBAMA. The seized meat was confiscated from the suspect’s freezer during routine surveillance activity (case 3). All three seizures were performed in the central-western region of Brazil, and species identifications were not possible from morphological data. Therefore, the cases could not be characterized as environmental crimes. To enable the appropriate law enforcement actions to be taken, we used the most widely used DNA markers for species identification, cyt-*b*[6] and COI [7]. DNA was obtained using a standard method of phenol-chloroform extraction and precipitation with ethanol [8]. A negative control was included in each DNA extraction process. A portion of about 1000 bp of cyt-*b* was amplified using the universal primers L14841 (5’AAAAAGCTTCCATCCAACATCTCAGCATGAAA3’) [9] and H15915 (5’AACTGCAGTCATCTCCGGTTTACAAGAC3’) [10]. The COI fragment (~700 bp) was amplified using the universal primers LCO1490 (5’ GGTCAACAAATCATAAAGATATTGG3’) and HC02198 (5’ TAAACTTCAGGGTGACCAAAAAATCA3’) [11]. The amplifications of both genes were carried out in a final volume of 30 μl containing 100 ng of DNA, 0.3 mM dNTPs, 1× PCR buffer (20 mM Tris–HCl pH 8.4, 50 mM KCl), 2.5 mM MgCl_2_, 2.5 U *Taq* DNA polymerase (Invitrogen) and 8 pmol of each primer. The PCR conditions were initiated by a denaturation at 94°C for 5 min, followed by 40 cycles at 94°C/30 s, 50°C/45 s, 72°C/45 s and a final extension at 72°C for 10 min. PCR contamination controls were performed. Each sample was sequenced twice on both strands using the Big Dye v3.1 Terminator Cycle Sequencing Kit (Applied Biosystems) in an ABI 3730 DNA Analyzer (Applied Biosystems). 

The sequences were manually edited using the software BioEdit Sequence Alignment Editor 7.0.9.0 [12]. This software was also used to align both strands and obtain consensus sequences for each sample. The sequences obtained were submitted as independent entries in a BLAST search for the most similar sequences using the default Megablast algorithm parameters [13]. For the species diagnosis, we considered the percentage homology between query and reference sequence pairs. In order to minimize the chance of incorrect species assignment, we adopted a threshold identity value of ≥98 % between the sequences [4]. Since this study aimed at the taxonomic identification of the seized meat samples, without any prior evidence or indication of the species, we regarded uploading the sequences to GenBank inadequate. The query sequences can be obtained directly from the authors.

The sequences of the seized samples were compared against those of species that were likely to be hunted or consumed at the seizure region (Table 1), which were downloaded from GenBank. When available, we used reference sequences of at least five different specimens of each species. The reference and suspected sequences were aligned using Clustal W implemented in BioEdit. Pairwise genetic distances were obtained according to the Kimura two-parameter model [14], and neighbor-joining analysis [15] was performed using Mega 3.1 [16]. Bootstrap values of the branch configuration in the trees were estimated using 1,000 replicates. 

**Table 1:** **Cyt-*b*reference sequences of species that would likely be hunted or consumed at the seizure region**

**Popular name**	**Species**	**GenBank accession numbers (ID reference sequence)**
Mammal species		
Indian cattle	*Bos indicus*	AY126697 (Bind1), EF693799.1 (Bind2), EF061238.1 (Bind3), EF061239.1 (Bind3), EF061242.1 (Bind4)
European cattle	*Bos taurus*	FJ971088 (Btau1), GU249572.1 (Btau2), DQ186288.1 (Btau3), DQ186284.1 (Btau4), AY952963.1 (Btau5)
Domestic pig	*Sus scrofa*	GQ351599 (Sscr1), GU135819.1 (Sscr2), AM492594.1 (Sscr3), AY237529.1 (Sscr4), GU135716.1 (Sscr5)
Spotted paca	*Cuniculus paca*	AY206574.1 (Cpac1), AY206563.1 (Cpac2), AY206572.1 (Cpac3), AY206561.1 (Cpac4), AY206560.1 (Cpac5)
Capybara	*Hydrochoerus hydrochaeris*	GU136721.1 (Hhyd1)
Marsh deer	*Blastocerus dichotomus*	DQ789176.2 (Bdic1), DQ789173.2 (Bdic2), DQ789175.2(Bdic3), DQ789174.2 (Bdic4)
Red brocket deer	*Mazama americana*	DQ789230.2 (Mame1), DQ789201.2 (Mame2), DQ789225.2 (Mame3), DQ789209.2(Mame4), DQ789224.2 (Mame5)
Gray brocket deer	*Mazama gouazoubira*	DQ789200.2 (Mgou1), DQ789189.2 (Mgou2), DQ789182.2 (Mgou3), DQ789203.2 (Mgou4), DQ789184.2 (Mgou5)
Pampas deer	*Ozotoceros bezoarticus*	L48404.1 (Obez1), DQ789199.2 (Obez2), DQ789198.2 (Obez3), DQ789191.2 (Obez4), DQ789192.2 (Obez5)
Collared peccary	*Pecari tajacu*	DQ179085.1 (Ptaj1), DQ179079.1 (Ptaj2), DQ179082.1 (Ptaj3), DQ179074.1 (Ptaj4), DQ179065.1 (Ptaj5)
White-lipped peccary	*Tayassu pecari*	AY534303.1 (Tpec1), U66290.1 (Tpec2), AY726775.1 (Tpec3)
Lowland tapir	*Tapirus terrestris*	AF056030.1 (Tter1), GQ259949.1 (Tter2), GQ259923.1 (Tter3), GQ259954.1 (Tter4), GQ259936.1 (Tter5)
Bird species		
Chicken	*Gallus gallus domesticus*	HQ122606 (Ggal1), AF195628.1 (Ggal2), AY029583.1 (Ggal3), AF354171.1 (Ggal4), AF028795.1 (Ggal5)
Red-throated piping-guan	*Aburria cujubi*	AY659799.1 (Acuj)
Blue-throated piping-guan	*Aburria cumanensis*	AY659798.1 (Acum)
Little tinamou	*Crypturellus soui*	FJ899152 (Csou1), FJ899151.1(Csou2), FJ899149.1 (Csou3), FJ899150.1 (Csou4), FJ899147.1 (Csou5)
Undulated tinamou	*Crypturellus undulatus*	AY139629.1 (Cund1)
Bare-faced curassow	*Crax fasciolata*	AY659790.1 (Cfas1), AY141923.1 (Cfas2)
Chaco Chachalaca	*Ortalis canicollis*/*Ortallis pantanalensis*	AF165472.1 (Ocan)/AY659783.1 (Opan)
Speckled Chachalaca	*Ortalis gutatta*	AY659782.1 (Ogut1)
Razor-billed curassow	*Pauxi tuberosa*	AY354484.1 (Ptub1), AF165469.1 (Ptub2)
Rusty-margined guan	*Penelope superciliaris*	AY659804 (Psup1)
Great tinamou	*Tinamus major*	AF338707.3 (Tmaj1)

The cyt-*b* sequencing of BIRD and mammal species (MAM1 and MAM2) produced readable sequences of approximately 550 and 1100 bp, respectively, which did not present insertions, deletions or stop codons. BLAST analysis of cyt-*b* indicated that the MAM1 meat sample was highly similar (99%) to the capybara (*Hydrochoerus hydrochaeris*) reference sequence, the MAM2 was similar (98%) to Pampas deer (*Ozotoceros bezoarticus*), and the BIRD sample was similar (98%) to Chaco chachalaca (*Ortalis canicollis*).

**Figure 2:**
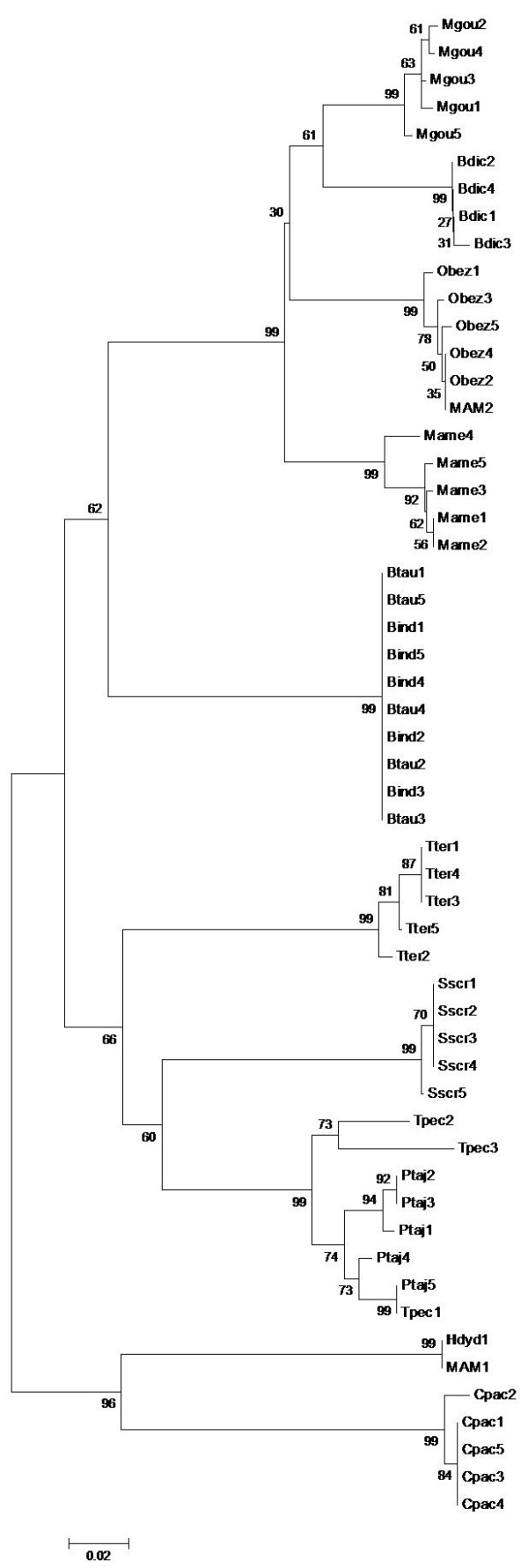
**Neighbor-joining tree of species that could likely have been consumed at the seizure region and MAM1 and MAM2 meat samples, based on the cyt-*b*molecular marker.**

**Figure 3:**
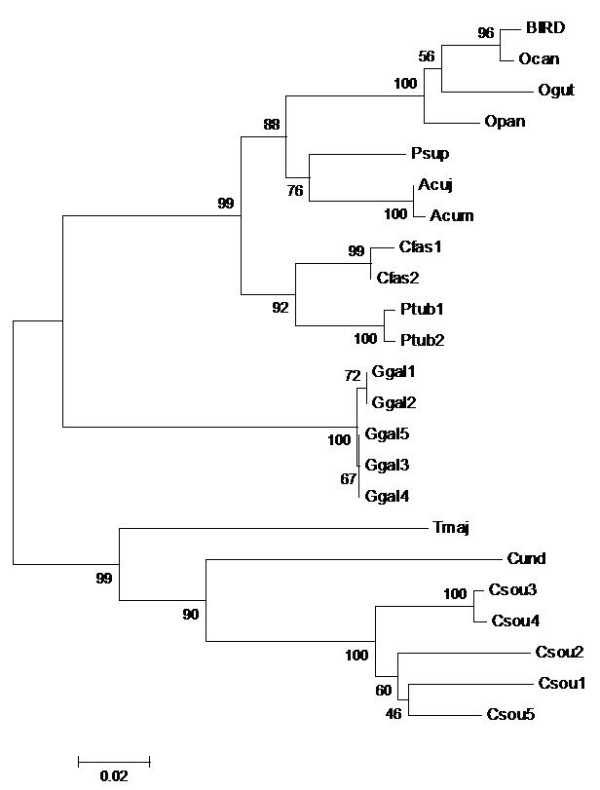
**Neighbor-joining tree of species that could likely have been consumed at the seizure region and BIRD meat sample, based on the cyt-*b*molecular marker.**

The comparative analysis using reference sequences of species that were likely to be hunted or consumed at the seizure region (Table 1) confirmed these results, as revealed by the genetic distance values and the neighbor-joining trees. MAM1 presented a genetic distance of zero when compared with the only capybara cyt-*b* reference sequence available in GenBank. MAM2 differed by 0.32% from the five Pampas deer specimens. The genetic divergence between the BIRD sample and the Chaco chachalaca was 0.9%. These genetic distance values were as low as those for the intraspecific genetic distances obtained for the species represented for more than one specimen reference sequence (average of 1.2% for mammals and 1.9% for birds), and lower than the cyt *b*-genetic distances (<2%) typical of population and intraspecific variation observed in mammal and bird species [17,18]. The species identifications of the seized samples were also supported by clades with 96-100% bootstrap values (Figures 2 and 3). Therefore, even with the questionable nature of the Genebank sequences, these results provide an indication of the species hunted. In a most conservative way, considering the family of the organisms hunted, the three cases already can be considered to be wildlife hunting crimes. 

Contrary to results with cyt-*b*, species identification was not possible using COI. The amplification of COI in BIRD, which could be solved using primers designed for bird species, was not successful. For the mammal species, the BLAST analysis did not match a reference sequence based in our threshold (≥98%). At the time of the manuscript writing, Genbank and BOLD did not contain COI reference sequences for capybara or for Pampas deer. Although the COI gene is considered the DNA barcoding gene [7], reference sequences for several species are still unavailable in these genetic databases. Researchers have used the cytochrome *b* gene for species identifications [5,19] since it is one of the better represented genes in GenBank [19] and has superior ability for separating species when compared with COI [20,21].

The three species killed are widely reported in hunting studies [1,22,23]. The capybara is widely distributed in South America and is the largest living rodent, weighing around 50 kg [23]. Pampas deer, the most endangered Neotropical cervid, formerly occupied a range of open habitats such as grassland, pampas and savanna (cerrado) in Brazil [24]. Its populations are decreasing because of habitat conversion for agriculture and cattle farming, hunting and attacks by feral dogs [24]. Its former range has been reduced to less than 1% [25]. The Chaco Chachalaca is a galliform commonly found in the Pantanal of Brazil. Cracid species are becoming rare because of hunting, and the loss and fragmentation of suitable habitats [22].

In this study the cyt-*b* molecular marker was suitable to diagnose the species killed and solve these criminal investigations. The suspected poaching in all three cases was confirmed with the identification of three wild species, capybara, Pampas deer and Chaco Chachalaca. In Brazil, hunting is a criminal offense, and based on the results of our molecular analysis, the defendants were found guilty and punished with fines; they may still be sentenced to prison for a period of 6 to 12 months.

## Abbreviations

COI: cytochrome *c* oxidase subunit I; cyt-*b*: cytochrome *b*; BOLD: Barcode of Life Database; mtDNA: mitochondrial DNA; IBAMA: Instituto Brasileiro do Meio Ambiente e dos Recursos Naturais Renováveis; MAM1: sample meat of the mammal seized in case 1; BIRD: sample meat of the bird species seized in case 2; MAM2: sample meat of the mammal seized in case 2; PCR: polymerase chain reaction.

## Competing interests

The authors declare that they have no competing interests.

## Authors’ contributions

AS coordinated and participated in the study design, carried out procedures in the laboratory, and drafted the manuscript. PMT, MSTG, CSC, CP and TGG helped in the DNA extractions, PCRs, data analysis and manuscript drafting. WAMP and FLN participated in the study design and helped to draft the manuscript. MG participated of the study conception and coordination, and helped to draft the manuscript. All authors read and approved the final manuscript.
